# Outcomes of Pregnancy in Patients With Chronic Liver Disease

**DOI:** 10.7759/cureus.72655

**Published:** 2024-10-29

**Authors:** Naeem Jan, Wiqas Ahmad, Zainab Zaib, Ayesha, Muhammad Ishfaq, Muhammad Kashif, Farooque Islam

**Affiliations:** 1 Gastroenterology and Hepatology, Hayatabad Medical Complex, Peshawar, PAK; 2 Obstetrics and Gynecology, Lady Reading Hospital, Peshawar, PAK; 3 Obstetrics and Gynecology, Indus Hospital and Health Network, Karachi, PAK; 4 Internal Medicine, Lady Reading Hospital, Peshawar, PAK; 5 Gastroenterology, Lady Reading Hospital, Peshawar, PAK

**Keywords:** chronic liver disease (cld), liver cirrhosis, pregnancy, pregnancy and chronic liver disease, pregnancy complication, pregnancy-related outcomes

## Abstract

Background and objectives

Chronic liver disease (CLD), due to its increased prevalence, is a global public health concern. With improved care of CLD patients nowadays, more patients with liver cirrhosis or CLD are getting pregnant. Pregnancy in the setting of CLD is associated with significant maternal and fetal complications. The objectives of this study were to determine the outcomes of pregnancy in patients with CLD and to ascertain the association between maternal CLD and pregnancy outcomes.

Materials and methods

This retrospective study was conducted at the Gastroenterology Department of Hayatabad Medical Complex, Peshawar, after approval from the hospital's ethical committee. A total of 91 pregnant patients with CLD were included. Data of these patients were retrieved from the hospital information management system. Baseline parameters and clinical parameters of the study participants were recorded. Maternal outcomes and fetal outcomes were noted. Data was analyzed using IBM SPSS Statistics for Windows, Version 25 (Released 2017; IBM Corp., Armonk, New York, United States). Inferential statistics, including univariate logistic regression analysis, was used to determine various factors influencing the fetomaternal outcomes. p-value ≤0.05 was considered statistically significant.

Results

The mean age of patients was 30.85±6.20 years, the mean gestational age was 29.66±4.15 weeks, and the mean CLD duration was 27.65±9.481 months. Viral hepatitis was the most frequent etiology of CLD (62.6%). Fifty-four patients (59.3%) were noted to have used fertility medications. Regarding maternal outcomes, 13 (14.3%) patients had preterm labor, 47 patients (51.6%) had a cesarean section as a mode of delivery, and post-partum hemorrhage (PPH) was observed in 13 (14.3%) patients. In terms of fetal outcomes, low birth weight was observed in 29 (31.9%) babies, intrauterine death (IUD) was noted in 13 (14.3%) babies and 27 babies (29.7%) had low Apgar scores at birth. The CLD duration was significantly associated with the mode of delivery (p<0.001). Similarly, maternal age (p=0.049), body mass index (p=0.017), and decompensated CLD (p=0.030) were associated with a high likelihood of IUD. Maternal age was associated with the likelihood of a low Apgar score (p=0.013).

Conclusion

Pregnancy in patients with CLD is associated with an increased risk of maternal complications (preterm labor and PPH) and fetal complications (IUD, low birth weight, and low Apgar score). Maternal age, body mass index, and decompensated CLD are associated with an increased likelihood of IUD.

## Introduction

Chronic liver disease (CLD) or liver cirrhosis results from persistent inflammation of hepatocytes, leading to scarring (fibrosis) and nodule formation [1]. CLD is a global public health concern with rising trends in incidence and prevalence [2]. Etiology is multifactorial. Depending on the geography, the pattern of etiologies varies, with alcohol as the leading cause in the Western population and chronic viral hepatitis in the Eastern population. The prevalence of CLD resulting from metabolic-associated liver disease is rising across the globe [3]. Apart from socio-financial implications, the disease is fatal and carries several complications. One and a half million patients reportedly died in the year 2019 as a result of CLD [4].

With background CLD, pregnancy is unlikely due to complex hormonal disturbances; however, with improvements in the care of CLD, more women are getting pregnant with concurrent liver cirrhosis [5,6]. Despite this, pregnancy in the setting of liver cirrhosis is high risk and carries several fetomaternal complications. At the same time, pregnancy may precipitate several CLD-related complications [7].

Adverse events were more frequently observed in pregnant and cirrhotic patients, including increased incidence of bleeding gums (7.2%), cesarean delivery (73.6%), precipitation of CLD-related complications like ascites (6.2%), and multi-organ failure (7.2%) in a case-control study [8]. Pre-term delivery and low birth weight occurred in higher proportions (19% and 15%, respectively) in another study of pregnant females with background CLD [9].

Being uncommon, the entity has seldom been studied. The majority of the information on pregnancy-related outcomes in the setting of CLD comes from case series studies, which are mainly conducted at international centers. Hence, the study has been planned to share the local experience with pregnancy outcomes in patients with background CLD.

## Materials and methods

Study design and settings

After approval from the Hospital Research and Ethical Committee (approval no: 2100), this retrospective study was carried out at the Department of Gastroenterology, Hayatabad Medical Complex, Peshawar. The study analyzed records of 91 pregnant females in the age range 20 to 40 years with background CLD, admitted during the period from 1st January 2022 to 30th June 2024. Patients with incomplete records, other medical conditions leading to fetomaternal complications like Rh incompatibility, post-liver transplant patients, patients with hepatocellular carcinoma, patients with overt hepatic encephalopathy, and patients with refractory variceal bleeding were excluded.

CLD was defined by the presence of both of the following: Serum albumin less than 3.5gm/dl and INR greater than 1.2. and ultrasound showing coarse nodular shrunken liver. Pregnancy outcomes that were assessed included maternal and fetal outcomes. Maternal outcomes included preterm labor, mode of delivery, and post-partum hemorrhage (PPH). Preterm labor was defined as labor onset before 37 weeks of gestation, mode of delivery was either normal vaginal delivery (NVD) or cesarean section (CS), while PPH was defined as blood loss >500ml in vaginal delivery and >1000ml in CS. Fetal outcomes that were assessed included intrauterine death (IUD), low birth weight (LBW), and Apgar score. IUD was defined as baby death within the uterus; a birth weight of a baby less than 2500 grams was considered as low birth weight, while Apgar (Appearance, Pulse, Grimace, Activity, and Respiration) score less than seven at five minutes after birth was considered as the low Apgar score.

Data collection

Patients' records were retrieved from the hospital management information system. Baseline information such as age, gestational age, parity, and BMI (body mass index) was recorded. Clinical factors like the etiology of CLD and disease duration were recorded. For maternal outcomes, the gestational age at the time of labor onset was noted to record preterm labor, followed by mode of delivery and amount of blood loss after delivery. Fetal outcomes that were recorded included IUD of the fetus, the weight of the baby at the time of delivery, and Apgar score at five minutes after birth.

Data analysis

Data was analyzed using IBM SPSS Statistics for Windows, Version 25 (Released 2017; IBM Corp., Armonk, New York, United States). Descriptive statistics was performed to present continuous data as mean and SD (standard deviation) and categorical data as frequencies and percentages. Inferential statistics included univariate logistic regression analysis, which was used to determine various factors influencing the fetomaternal outcomes. p-value ≤0.05 was considered statistically significant.

## Results

As reported in Table 1, the mean age of the patients ranged from 20 to 40, with a mean age of 30.85±6.20 years.

**Table 1:** Mean and standard deviation of patients according to various parameters (n = 91) BMI: Body mass index, CLD: Chronic liver disease

Parameters	Minimum	Maximum	Mean	Standard Deviation
Age (years)	20	40	30.85	6.200
Gestational age (weeks)	22	36	29.66	4.159
BMI (kg/m^2^)	21.0	25.6	23.613	1.1540
CLD duration (months)	10	45	27.65	9.481

Chronic viral hepatitis was the most frequent etiology of CLD recorded in 57 participants (62.6%). Fifty-four patients (59.3%) used fertility medications for conception. Regarding the severity of CLD, 23 patients (25.3%) had decompensated CLD as shown in Table 2.

**Table 2:** Frequencies and percentages of patients according to various socio-clinical parameters (n = 91) BMI: Body mass index, CLD: Chronic liver disease, PBC: Primary biliary cholangitis, BCS: Budd-Chiari syndrome

Parameters	Subgroups	Frequency	Percentage
Age (years)	30 or below	42	46.2
	More than 30	49	53.8
Gestational age (weeks)	30 or below	47	51.6
	More than 30	44	48.4
Parity	Primiparous	22	24.2
	Multiparous	69	75.8
BMI (kg/m^2^)	23.0 or below	32	35.2
	More than 23.0	59	64.8
CLD duration (months)	20 or below	23	25.3
	More than 20	68	74.7
Etiology	Viral	57	62.6
	Autoimmune	6	6.6
	PBC	9	9.9
	BCS	6	6.6
	Idiopathic	13	14.3
Conception	Spontaneous	28	30.8
	Medications	54	59.3
	Assisted techniques	9	9.9
Booking status	Booked	73	80.2
	Unbooked	18	19.8
CLD	Decompensated	23	25.3
	Compensated	68	74.7

Maternal outcomes are reported in Table 3. Both preterm labor and PPH occurred in 13 (14.3%) of patients, while the predominant mode of delivery was CS.

**Table 3:** Frequencies and percentages according to maternal outcomes (n = 91) NVD: Normal vaginal delivery, CS: Cesarean section, PPH: Post-partum hemorrhage

Maternal outcomes	Subgroups	Frequency	Percentage
Preterm labor	Yes	13	14.3
	No	78	85.7
Mode of delivery	NVD	44	48.4
	CS	47	51.6
PPH	Yes	13	14.3
	No	78	85.7

Fetal outcomes are summarized in Table 4. IUD was recorded in 13 participants (14.3%), and 29 babies (31.9%) had low birth weight.

**Table 4:** Frequencies and percentages according to fetal outcomes (n = 91) IUD: Intrauterine death, LBW: Low birth weight, Apgar score: (Appearance, Pulse, Grimace, Activity, and Respiration) score

Fetal outcomes	Subgroups	Frequency	Percent
IUD	Yes	13	14.3
	No	78	85.7
LBW	Yes	29	31.9
	No	62	68.1
Low Apgar	Yes	27	29.7
	No	64	70.3

As reported in Table 5, none of the independent factors influencing preterm birth had a p-value <0.05. Hence, the factors did not add significantly to the model.

**Table 5:** Logistic regression analysis for factors influencing preterm labor (n = 91) S.E: Standard error, B: Beta coefficient, Exp(B): Exponentiated beta coefficient, CLD: Chronic liver disease, Hyphen (-) indicates an empty cell which means that the model could not compute an upper bound for the odds ratio Exp(B).

Factors	B	S.E.	Wald	p-value	95% C.I for Exp(B)	
					Lower	Upper
Age (years)	-0.095	0.089	1.142	0.285	0.764	1.082
Gestational age (weeks)	-0.047	0.122	0.151	0.698	0.751	1.211
BMI (kg/m^2^)	0.295	0.555	0.282	0.595	0.453	3.982
Disease duration (months)	0.081	0.056	2.070	0.150	0.971	1.210
Etiology (viral)	-20.301	8706.285	0.000	0.998	0.000	-
Conception (spontaneous)	-18.630	12518.595	0.000	0.999	0.000	-
Booking status (booked)	2.512	1.557	2.603	0.107	0.583	260.973
CLD (decompensated)	-22.086	6893.089	0.000	0.997	0.000	-

The p-value for the independent factor, CLD duration, was 0.001, which was <0.05 as shown in Table 6. The number of months of CLD was associated with the mode of delivery and adds significantly to the model.

**Table 6:** Logistic regression analysis for factors influencing the mode of delivery (n = 91) S.E: Standard error, B: Beta coefficient, Exp(B): Exponentiated beta coefficient, CLD: Chronic liver disease, Hyphen (-) indicates an empty cell which means that the model could not compute an upper bound for the odds ratio Exp(B), p-value with an asterisk (*) indicates that the value for that parameter is <0.05 and is significant.

Factors	B	S.E.	Wald	p-value	95% C.I for Exp(B)	
					Lower	Upper
Age (years)	-0.105	0.054	3.804	0.051	0.811	1.001
Gestational age (weeks)	0.070	0.077	0.832	0.362	0.922	1.248
BMI (kg/m^2^)	-0.559	0.296	3.579	0.059	0.320	1.020
Disease duration (months)	-0.131	0.040	11.011	0.001*	0.812	0.948
Etiology (viral)	-1.197	0.938	1.629	0.202	0.048	1.899
Conception (spontaneous)	-22.354	12241.882	0.000	0.999	0.000	-
Booking status (booked)	-0.728	0.967	0.567	0.452	0.073	3.213
CLD (decompensated)	-0.023	0.769	0.001	0.976	0.216	4.412

As shown in Table 7, none of the studied independent factors for post-partum hemorrhage had a p-value <0.05. Hence, the factors did not add significantly to the model.

**Table 7:** Logistic regression analysis for factors influencing post-partum hemorrhage (n = 91) S.E: Standard Error, B: Beta Coefficient, Exp(B): Exponentiated Beta Coefficient, CLD: Chronic Liver Disease, Hyphen (-) indicates empty cell which means that the model could not compute an upper bound for the odds ratio Exp(B)

Factors	B	S.E.	Wald	p-value	95% C.I for Exp(B)	
					Lower	Upper
Age (years)	-0.232	0.128	3.265	0.071	0.617	1.020
Gestational age (weeks)	0.152	0.106	2.060	0.151	0.946	1.433
BMI (kg/m^2^)	-1.579	0.723	4.767	0.029	0.050	0.851
Disease duration (months)	-0.012	0.066	0.033	0.855	0.869	1.124
Etiology (viral)	1.766	1.305	1.831	0.176	0.453	75.423
Conception (spontaneous)	-19.047	12687.816	0.000	0.999	0.000	-
Booking status (booked)	0.141	1.513	0.009	0.926	0.059	22.354
CLD (decompensated)	1.832	1.552	1.394	0.238	0.299	130.728

As illustrated in Table 8, the p-values for independent factors, maternal age, BMI, and decompensated CLD were all found to be <0.05, indicating their significant influence on the dependent factor, intrauterine death.

**Table 8:** Logistic regression analysis for factors influencing intrauterine death (n = 91) S.E: Standard error, B: Beta coefficient, Exp(B): Exponentiated beta coefficient, CLD: Chronic liver disease, p-value with an asterisk (*) indicates that the value for that parameter is <0.05 and is significant.

Factors	B	S.E.	Wald	p-value	95% C.I for Exp(B)	
					Lower	Upper
Age (years)	0.344	0.175	3.866	0.049*	1.001	1.988
Gestational age (weeks)	-0.789	0.446	3.133	0.077	0.189	1.088
BMI (kg/m^2^)	1.689	0.710	5.663	0.017*	1.347	21.755
Disease duration (months)	0.112	0.073	2.355	0.125	0.970	1.290
Etiology (viral)	0.959	1.198	0.641	0.423	0.249	27.286
Conception (spontaneous)	1.652	1.268	1.697	0.193	0.434	62.691
Booking status (booked)	-4.207	2.530	2.765	0.096	0.000	2.121
CLD (decompensated)	5.464	2.523	4.688	0.030*	1.679	33171.479

The influence of the studied independent factors on the likelihood of low birth was statistically insignificant, as evident from the p-value>0.05, shown in Table 9.

**Table 9:** Logistic regression analysis for factors influencing low birth weight (n = 91) S.E: Standard error, B: Beta coefficient, Exp(B): Exponentiated beta coefficient, CLD: Chronic liver disease

Factors	B	S.E.	Wald	Sig.	95% C.I for EXP(B)	
					Lower	Upper
Age (years)	-0.036	0.050	0.522	0.470	0.875	1.064
Gestational age (weeks)	0.006	0.065	0.008	0.931	0.886	1.142
BMI (kg/m^2^)	0.080	0.237	0.114	0.736	0.681	1.723
Disease duration (months)	0.012	0.034	0.120	0.729	0.946	1.082
Etiology (viral)	-1.005	0.908	1.226	0.268	0.062	2.169
Conception (spontaneous)	0.378	0.963	0.154	0.695	0.221	9.640
Booking status (booked)	-1.635	0.986	2.752	0.097	0.028	1.346
CLD (decompensated)	-0.974	0.730	1.780	0.182	0.090	1.579

Maternal age in years was associated with the likelihood of a low Apgar score, as shown in Table 10.

**Table 10:** Logistic regression analysis for factors influencing a low Apgar score (n = 91) S.E: Standard error, B: Beta coefficient, Exp(B): Exponentiated beta coefficient, CLD: Chronic liver disease, p-value with an asterisk (*) indicates that the value for that parameter is <0.05 and is significant.

Factors	B	S.E.	Wald	Sig.	95% C.I for EXP(B)	
					Lower	Upper
Age (years)	-0.137	0.055	6.207	0.013*	0.782	0.971
Gestational age (weeks)	-0.002	0.066	0.001	0.971	0.877	1.134
BMI (kg/m^2^)	-0.331	0.256	1.671	0.196	0.434	1.187
Disease duration (months)	0.001	0.034	0.001	0.972	0.937	1.069
Etiology (viral)	0.299	0.747	0.160	0.689	0.312	5.826
Conception (spontaneous)	0.821	1.039	0.625	0.429	0.297	17.429
Booking status (booked)	0.578	0.891	0.421	0.517	0.311	10.230
CLD (decompensated)	-0.509	0.644	0.623	0.430	0.170	2.126

## Discussion

The mean age of the participants in this study was 30.85±6.20 years. The mean age of pregnant patients with cirrhosis was 30.79±5.01 years compared to 31.38±4.13 in patients without cirrhosis in a study by Gao et al., which is quite similar to our observation [8]. The median maternal age in patients with cirrhosis was 31 (27-34) years in another study [10]. In another study, the mean maternal age in patients with cirrhosis was 31.88 ± 5.82 years compared to 30.93 ± 4.03 years in the control group (without cirrhosis) [11]. Though the mean age in our cohort was similar to the results of the previous studies, in the absence of a control group in this study we could not draw any comparison with the general population.

The mean gestational age in our study was 29.66±4.15 weeks. The mean gestational age in a study by Tan et al. was 33.38 ± 3.78 weeks [11]. The mean gestational age in their study was slightly higher than ours. This difference may be due to a difference in the severity of CLD. All of the patients in the later study had decompensated cirrhosis, compared to 23 (25.3%) in our study. Chronic viral hepatitis was the frequent cause of CLD in our study, observed in 57 patients (62.6%). In another study, chronic viral infections were most frequently reported among pregnant women presenting to our center. HCV was reported in 30.3% of patients, and HBV was observed in 11.5% of patients [12]. Similar findings were reported in another study [10].

Preterm labor was recorded in 13 (14.3%) patients in our study. In a study by Huang and colleagues, the percentage of preterm births in CLD mothers was 6.9%, which was lower than our observation [13]. The odds ratio for occurrence of preterm birth was 3.1 95% (CI 1.9-4.9). However, the authors in the later study differentiated patients with advanced liver cirrhosis from minimal cirrhosis as opposed to our study, where all patients with slight evidence of liver cirrhosis were considered for the same group. In another study, the percentage of preterm delivery in the cirrhotic group was 25.0%, much higher than our observations [14].

Forty-seven patients (51.6%) in our study had a cesarean delivery, and 44 (48.4%) had NVD. However, there are specific recommendations about the mode of delivery in CLD patients [15]. Gao et al. reported a higher incidence of cesarean delivery in CLD patients as compared to the general population (73.6%. vs. 49.5%) [8]. The mode of delivery for CLD patients is managed on a case-to-case basis. CS is usually performed when there is a high likelihood of fetal distress during attempted labor or when there is a prior history of CS. However, CLD alone does not indicate CS [16].

PPH was observed in 13 patients (14.3%). Bleeding in CLD patients is challenging. The pathophysiology is multifactorial. It may be due to portal hypertension or thrombocytopenia [17]. In such circumstances, the risk of post-partum bleeding is further increased in pregnant patients [18]. In a study by Anjum et al., PPH was recorded in 10.5% of cases, slightly lower than our observation, while Jena et al. reported PPH in 17.5% of patients [19,20]. IUD was recorded in 13 patients (14.3%). Gaikwad et al. concluded that the higher proportion of fetal loss in pregnancy with background liver disease is attributed to liver dysfunction. The proportion of stillbirths in their study was 4.0% [21]. The proportion of fetal demise at various intervals during and after gestation was higher, as reported in a study by Jena et al. In their study, fetal loss prior to the age of viability was 34.0%, and stillbirths occurred in 6% of cases [20].

Low birth weight was observed in 29 (31.9%) patients. Salman et al. showed that the incidence of low birth weight was significantly higher in patients with cirrhosis than in the control group (25.0% versus 7.0%) [14]. In another study by Gao et al., the proportion of low birth weight in the cirrhotic group was 13.6% compared to 2.6% in the control group [8].

Although our study is the first comprehensive study to provide local literature on this important topic, it has few limitations. First, it was a single-center study, which limits the generalizability of its results. Second, it was a retrospective study. Lastly, the sample size was small, and the absence of a control group was also a significant limitation.

## Conclusions

Pregnancy in patients with CLD is associated with significant fetomaternal complications. In our study, preterm labor and PPH were the most frequent maternal complications. The most common mode of delivery in our study participants was CS. The most frequent fetal complications in our study were low birth weight followed by a low Apgar score at birth and IUD. Maternal age, body mass index, and decompensated CLD are associated with an increased likelihood of IUD. Since patients with CLD are at increased risk of adverse fetomaternal outcomes, therefore they should be followed and monitored more closely.
